# Lab protocol using framework analysis to capture practitioners’ perspectives on the usability of virtual reality for sport rehabilitation

**DOI:** 10.1371/journal.pone.0337814

**Published:** 2025-12-30

**Authors:** Hannah K. M. Tang, Mark J. Lake, Frederic A. Bezombes

**Affiliations:** 1 School of Engineering, Liverpool John Moores University, Liverpool, United Kingdom; 2 Research Institute for Sport and Exercise Sciences, Liverpool John Moores University, Liverpool, United Kingdom; Soochow University, CANADA

## Abstract

Addressing issues in sport rehabilitation with technology-based applications is becoming more common due to improved technological solutions. This drives the need for evidence-based rationales and decisions; technology features should be informed by expert opinion and practice. The current lab protocol paper proposes the use of the framework analysis (FA), a qualitative data analysis method, for identifying practitioners’ perspectives on technology solutions aimed at aiding athlete rehabilitation. The FA allows data to be analysed in a structured and rigorous process, whilst also allowing for the flexibility associated with qualitative enquiry. Subsequently, this method is being increasingly used in healthcare and nursing research and could be applied in the context of using technology to enhance sport rehabilitation. In the current paper, FA is applied in the context of determining the usability of virtual reality (VR) in sport rehabilitation and obtaining perspectives on VR features necessary for integration into existing rehabilitation practice. The paper includes a worked example, taking it from raw data to a working theme. The use of the current lab protocol led to the identification of interconnected key themes regarding the following VR application features: how the VR application would be delivered, what the VR application would involve, and why, where, and when the VR application would be used. The lab protocol also allowed subthemes to be derived, indicating how these VR features would be met. The findings inform the ongoing development of a VR application designed to assess quick directional change in sports, potentially applied to the treatment of musculoskeletal injury in athletes. The use of the FA to derive this content is susceptible to limitations present in all qualitative data processing, such as reflexivity, the implications of levels of rigour, as well as being a time-consuming process.

## 1 Introduction

Return to sport after injury can come with a variety of associated costs for many stakeholders. These may include psychological, social, financial and physical costs, with athletes facing the greatest impact [1–3]. The variety of technology-based applications developed to address issues in sport rehabilitation is growing due to the increased accessibility of improved technological solutions [4]. This drives the need for evidence-based rationales and decisions; technology features should be informed by expert opinion and practice [5]. Without a clear or valid basis for application or tool development, the technology produced may not meet the needs of the end user. This could render the technology not fit for purpose or could affect the delivery of the application and the integration of the final product into current sport rehabilitation practice [5].

Whilst recent research has begun exploring practitioner and athlete perspectives on virtual reality (VR) in sport rehabilitation, this is limited as it is an emerging field. The review of existing literature demonstrates a considerable body of evidence supporting the effectiveness of VR interventions in sporting rehabilitation. In particular, the studies highlight improved patient recovery, reflected in enhanced functional abilities and increased muscular strength [6]. However, the literature does not explicitly state whether these have been informed by practitioner perspectives and practice. Few studies document input from practitioners to shape application development. In one such example, Gouveia et al. [7] informed the development of their VR system for musculoskeletal sporting injuries by interviewing physiotherapists working in professional football (soccer). A qualitative analysis of these interviews produced a validated list of rehabilitation exercises, later translated into a football-themed VR environment. The system was then tested with 37 university students from diverse fields, who completed a full VR rehabilitation session across five customised games. Data collection included physiological measures (heart rate, perceived exertion) with subjective assessments obtained through a post-session survey.

The survey design (Gouveia et al. [7]) provided a multidimensional view of participants’ subjective experiences with VR rehabilitation. The System Usability Scale (SUS), a widely used 10-item questionnaire, measured usability by asking participants to rate ease of use, clarity, and overall system functionality. To capture psychological engagement, selected items from the Intrinsic Motivation Inventory (IMI) were used, focusing on dimensions such as enjoyment, perceived competence, and effort, which reflect how motivating participants found the VR session. Dedicated questions on immersion (Immersive Tendencies Questionnaire) and presence (Witmer−Singer Presence Questionnaire) assessed the extent to which users felt absorbed in the virtual environment. However, they did not use open-ended questions for participants to provide in-depth reflections on their experiences, enabling them to highlight valued aspects, difficulties encountered, or suggestions for improvement.

In contrast, Lewellen et al. [8] explored VR adoption from the athlete’s perspective. Fourteen athletes with VR experience, most of whom competed at the collegiate level, were recruited to participate in the study. Data was collected through semi-structured interviews conducted via Zoom, which were audio-recorded and transcribed verbatim. Analysis utilised Braun and Clarke’s six-phase reflexive thematic framework, combining inductive coding with deductive elements informed by the Technology Acceptance Model and the “4Ws” framework (where, when, why, what). This process produced nine overarching themes that captured athlete perceptions, including enthusiasm for VR, recognition of barriers such as cybersickness, cost, and coach attitudes, and suggestions for strategies to facilitate broader use.

The first-hand accounts that were captured by Lewellen et al. [8] were analysed through reflexive thematic analysis. Their use of Braun and Clarke’s inductive thematic analysis and the deductive method of the Technology Acceptance Model provided a means of exploratory investigation as well as structure. A combination of standardised scales and open-ended feedback could offer a robust evaluative framework that assesses not only the technical performance of the VR system but also its psychological and experiential impact on users. Building on this work, qualitative and in-depth Framework Analysis (FA) can incorporate existing rehabilitative practices and the perspectives of practitioners. This can be used to highlight both the immersive benefits of VR and the practical obstacles to its adoption.

### 1.1 Theoretical and conceptual framework

The FA method, originally developed by U.K. social policy researchers as a practical tool for real-world investigations [9], has since been widely applied to specific research questions, particularly in healthcare and nursing, and holds promise for sport rehabilitation. FA is well suited to managing large datasets generated from interviews, focus groups, or case studies, offering a structured yet flexible approach to qualitative enquiry [10,11]. This makes it ideal for use with a purposive sample, where the findings are not necessarily representative of all rehabilitation contexts but provide ecologically valid insights from individuals in the most suitable positions [10].

Compared to other qualitative methods, FA offers distinct advantages that directly address common limitations seen in other qualitative data analysis methods. While thematic analysis (TA) is widely appreciated for its flexibility, its open-ended nature can compromise transparency, as coding criteria may be inconsistently documented, interpretations can vary significantly between researchers, and the analytical path from raw data to conclusions is often difficult to audit or reproduce [12]. Grounded Theory (GT), though valuable for generating new theories, is highly inductive and time-consuming, making it less suitable for studies with predefined questions or practical constraints [13]. Likewise, methods such as Interpretative Phenomenological Analysis (IPA), narrative analysis, and discourse analysis provide rich, nuanced insights into individual experiences and language use, but they tend to lack scalability and are less compatible with structured evaluations or applied research settings [14].

In contrast, FA integrates the benefits of different methods. Data can be described in the participants’ own subjective expressions prior to interpretation and before being summarised systematically and clearly in a matrix, allowing meaning to be preserved while making the dataset manageable [10,15]. The method integrates both deductive and inductive reasoning, enabling researchers to draw on existing knowledge and an overarching question, whilst also generating theories from observed patterns [10]. This dual approach makes FA particularly relevant for VR in rehabilitation, where documented rehabilitation practices can be combined with participant perspectives to inform application design. The process is iterative and recursive, involving continual reference to transcripts to ensure coding accuracy and authentic representation of participants’ views [9,15]. Through this, themes and subthemes can be identified, mapped, and interpreted for practical application [16].

In this study, FA is used to determine the usability of VR and identify the core features required for integration into sport rehabilitation, with findings informing the development of a VR application for assessing rapid directional change and supporting musculoskeletal injury treatment in athletes. By combining methodological rigour with practical relevance, FA emerges as the most appropriate and effective framework.

This paper proposes the use of the qualitative data analysis method FA to analyse interview data of rehabilitation specialists in order to answer the question, ‘What are the core features required for a virtual reality (VR) application to enhance sport rehabilitation?’. The method could therefore determine perceptions on usability of VR and obtain perspectives on VR features necessary for its application into existing sport rehabilitation. This protocol could be applied in any context to capture the expertise of specialists and end users to inform the development of a VR application.

**Fig 1:**
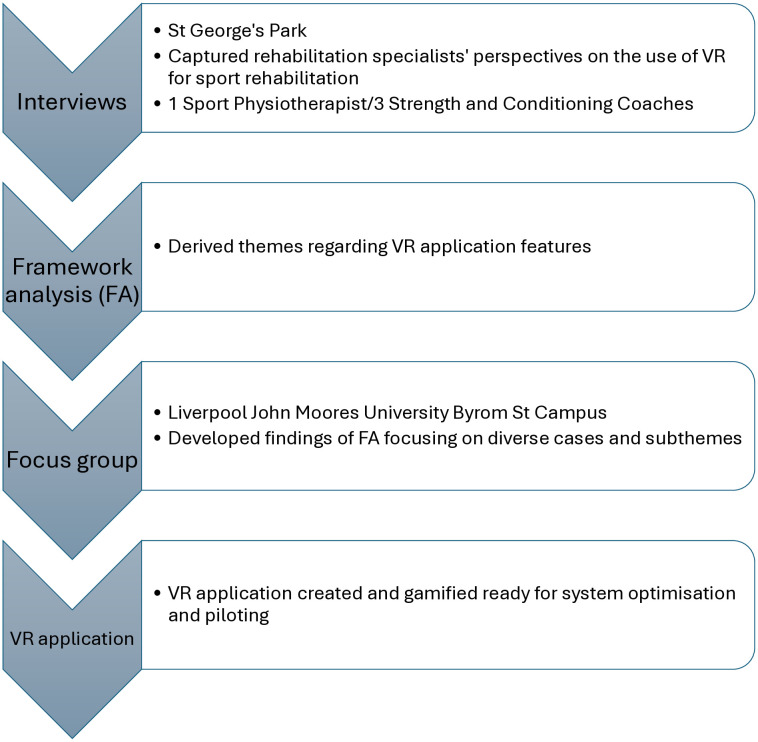
Visual flowchart of current stages to main study.

The current paper forms part of a larger study with the following objectives (see Fig 1 for a visual flowchart of the main study’s current stages): 1) observe the use of instrumented equipment employed in a sport rehabilitation context, 2) conduct interviews with one Sport Physiotherapist and three Strength and Conditioners, 3) apply the FA method for processing qualitative data, 4) conduct a focus group to widen the input from practitioners with varied backgrounds including VR-rehabilitation research, 5) use the guidance on the VR application’s implementation and required benefits for RTS to inform its development. The main objective of the current lab protocol was to utilise a worked example, illustrating how the FA process was conducted and the themes reached, specifically pertaining to objectives 3, 4 and 5 of the larger study. The findings have informed the ongoing development of a VR application designed to assess quick directional change in sports. For a review of existing VR applications and how these inform the novel features proposed, as well as a development and pilot of the VR application, see Tang et al. [17].

## 2 Materials and methods

### 2.1 Ethics

Prior to commencement, the study received ethical clearance from Liverpool John Moores University Research Ethics Committee; reference number: 20/EEE/001. On commencing the interviews, individuals were provided with participant information forms and they provided written consent; this also ensured a uniform introduction to data collection. The forms provided researchers with information that could potentially identify participants, and the forms were kept in a secure location, with all further data coded to protect participant identification. The codes were stored separately from the forms and data to preserve anonymity.

### 2.2 Associated content

The protocol described in this peer-reviewed article is published on protocols.io, https://dx.doi.org/dx.doi.org/10.17504/protocols.io.e6nvw8r82vmk/v1 [Accessed: 20/11/2025] and is included for printing as S1 File with this article.

### 2.3 Duration

This study was conducted from the 18^th^ Feb. 2020–31^st^ Jun. 2022. Communication to request permission to conduct the observations and interviews, and for a discussion on recruitment, occurred via email on 18^th^ Feb. 2020. The interviews were held over one day at St George’s Park [18] on 6^th^ Mar. 2020. Final correspondence with the interview participants occurred via email on 25^th^ Mar. 2021. Auditing of categories with osteopath and biomechanist occurred at Liverpool John Moores University Byrom Campus on 26^th^ Nov. 2021 and 22^nd^ May 2022. The focus group recruitment occurred on 26^th^ May 2022. The focus group occurred on 31^st^ May 2022. Auditing of quotations occurred with English language specialist on 6^th^ Jun. 2022.

## 3 Sample data, results and discussion

**Fig 2:**
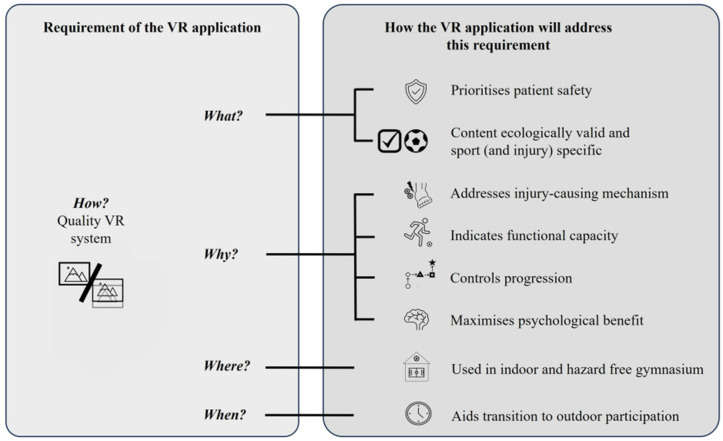
Key themes and their relevance to VR usability.

The interview study identified four main themes of what, why, where and when. A summary of the key themes and their relevance to VR usability is depicted in Fig 2.

The sample data below is a worked example of how this protocol was applied in order to arrive at the themes. For further advice on how to interpret and analyse the data, see the step-by-step protocol (S1 File) where it is explained by stage and in full. The example included in this paper follows the progression of one theme: when to implement the VR application. The example demonstrates the process from raw data to theme formation and visualisation.

The data processing was originally conducted in a combination of various formats: hard copy (initial thoughts), NVivo (coding), Excel (framework), Inspiration 10 (mind mapping software), PowerPoint (presentation/diagrams of final themes and sub-themes), and Word (write-up). As explained in the step-by-step protocol (S1 File), the software format selected was based on what was optimal for that specific stage of processing. For example, a hardcopy format increased ease of reading; a primary feature of NVivo was the ability to organise and code data by participant with ease; Excel offered a matrix for tabulating data; Inspiration offered a solution for visualising categories and allowing connections, similarities and differences to be visually presented to aid the formation of themes; PowerPoint allowed for easy manipulation of objects; Word was most suitable for writing-up findings. Subjectively, transferring output from one system to another was deemed to be simple; however, if analysts determine that a particular software programme is not personally advantageous, then they should select those which they consider to be most convenient.

For the example data below, this has been collated in Word for clarity, i.e., one can see the data processing methods taken at each stage and compare the stages in order to understand how the process and output developed. Additionally, the following data is not definitive nor conclusive (dedicated publications will present final output). See Table 1 for definitions as applied in the context of this worked example [10].

**Table 1:** Definitions as applied to this worked example.

Term	Definition
Analytical framework	A set of codes organised into categories used to organise the data, potentially arranged in a tree diagram structure. The framework creates a structure for the data that is helpful to summarise/reduce the data to answer the research question(s).
Categories	Codes are grouped into clusters around similar and interrelated ideas or concepts to help form categories, which are closely and explicitly linked to the raw data.
Code	A descriptive or conceptual label assigned to excerpts of raw data in a process called ‘coding’.
Data	Texts, produced by transcribing interview data or creating ‘field’ notes while conducting participant-observation or observing objects or social situations.
Indexing	The systematic application of codes from the analytical framework to the whole dataset.
Matrix	A spreadsheet containing numerous cells into which summarised data are entered by codes (columns) and participants (rows).
NVivo	Qualitative data analysis software tool that allows, amongst other features, for data to be organised, coded, and categorised with ease.
Theme	Interpretive concepts or propositions that describe or explain aspects of the data, which are the final output of the analysis of the whole dataset. Themes are articulated and developed by interrogating data categories through comparison between and within participants. Usually, a number of categories would fall under each theme or sub-theme.
Thematic network	Mind-map-like illustrations that summarise the main themes.
Transcript	A written word-for-word account of an interview.

**Fig 3:**
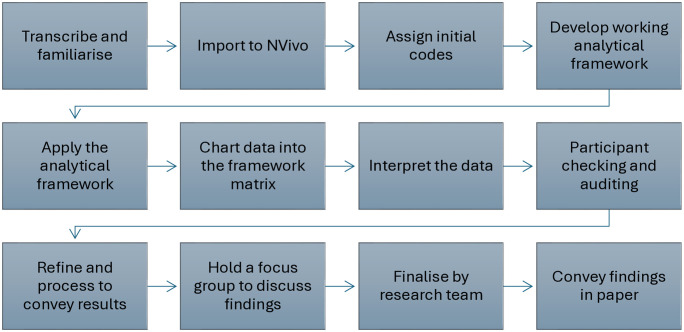
Visual flowchart summarising the protocol.

Table 2 identifies the stages of data processing. The stages required to produce the data samples can be seen in the step-by-step protocol (S1 File), alongside a detailed explanation of each data processing step. This is also conveyed in the visual flowchart summarising the protocol (Fig 3).

**Table 2:** Stages to data processing.

Stage of data processing	Purpose of stage	Activity
A. Transcribe and familiarise	Record data in written form for initial processing and reviewing to increase familiarity.	Transcribe recording on Microsoft Word.
		Read transcripts and hand-written notes made during interviews. Check accuracy of transcripts.
		Write down ideas that are reflective and important on hand-written notes.
B. Import to NVivo	Place data in analysis software tool for ease of processing.	Organise data by interviewee on NVivo.
C. Assign initial codes	Break data down into smaller segments and assign labels (codes) for systematic comparison between datasets.	Identify initial expected codes via deductive coding using research question, interview topics and important ideas noted during and after interviews on NVivo.
		Assign deductive codes to all data systematically on Nvivo.
		Code data inductively on NVivo open to as many different perspectives of end users as possible, e.g., athletes, exercise physiotherapists, strength and strength and conditioners etc.
		*Second coder with experience in qualitative research, blind codes 12 randomly selected data excerpts inductively on Microsoft Excel. Primary researcher and second coder discuss initial and potential codes with rationale and justifications.
D. Develop working analytical framework	Identify similarities between codes and place into groups accordingly. This allows for further organisation of data into an initial analytical framework.	Form initial analytical framework by grouping codes into categories on NVivo. In this way, categories are defined.
		Label data that is not easy to categorise as ‘other’.
E. Apply the analytical framework	Place codes for all transcripts into the developing analytical framework (indexing) to solidify categories. Code any unlabelled data to expand categories.	Apply the analytical framework by indexing transcripts, using existing categories and codes.
		Code all ‘other’ data placing ‘other’ data into existing coding index and/or expanding coding index with the emergent codes (Microsoft Excel).
F. Chart data into the framework matrix	Summarise and tabulate data for later processing into themes. This facilitates the emergence of themes of more abstract concepts that represent recurring patterns or ideas across the coded data.	Summarise coded data by category with quotations and place summaries in a further spreadsheet matrix (Microsoft Excel).
G. Interpret the data	Develop and refine distinct themes, with visual mind-mapping representation.	Identify characteristics and differences of data to form themes.
		Refine themes to develop the thematic network map using Inspiration 10 mind-mapping software.
H. Participant checking and auditing	Ensure participant perspectives are accurately conveyed and underlying meanings are captured through suitable quotations and themes.	Maintain dialogue with interviewees and ensure representative quotations were approved by interviewees.
		Themes audited: discuss map and ideas with auditing biomechanist and osteopath to review themes.
I. Refine and process to convey results	Finalise thematic network and separate to visually represent themes and their relationships, highlighting how they connect and interact with each other.	Separate thematic network by theme in order to transfer themes and subthemes from Inspiration 10 to PowerPoint and then to Microsoft Word. Attribute icons to represent links between themes. Tabulate information.
		*Osteopath audited diagrams and table.
		Define and name themes. Researchers agree to finalise themes and subthemes.
		Hold a focus group to address diverse cases and subthemes in which the primary researcher would not otherwise have knowledge or experience.
		Check themes relate to coded extracts. Quotes audited against data set by language specialist.
		Finalised by research team. Paper produced.

*Dependent on expertise available.

### 3.3.1 Transcribe, familiarise, import to NVivo and assign codes.

After the data was transcribed and read, important ideas were written on the interview notes and data was organised by interviewee on NVivo.

### 3.3.2 Assign initial codes.

The following are examples of raw data extracts relevant to the final theme ‘When’. This is not an exhaustive list and is merely for illustrative purposes to demonstrate how the step-by-step process is applied: i.e., many more codes contributed to the eventual formation of this theme.

Raw data extracts (and further surrounding text for context) were highlighted in NVivo by line. Samples of raw data excerpts are seen in Table 3, with corresponding examples of initial assigned codes. ‘Over-coding’ was favoured to allow for a more thorough interpretation and some of these initial codes will have been merged on review. Conversely, more codes may have been added on re-reading the data in context at a later stage. Additionally, these codes will have also contributed to the formation of different categories and other final themes.

**Table 3:** Example data extracts and preliminary coding, SP = Sport Physiotherapist; SC = Strength and Conditioner.

Interviewee	Raw Data Extract	Corresponding Initial Code
SP	‘You’re looking to get to a stage where you clear up all the basics, so you make sure you have what we term ‘the ankle is healthy’: there is no swelling, there is no pain, there is full movement, and there is isolated strength. And then once they’ve got that, and they are functionally day-to-day pain-free, we move onto the sport specific stuff, which I term as tick box, basically, set them an activity, can they do it?’	The ankle is healthy, primarily return to functional state, stages, phases of recovery, functionality before sport-specific activity, clearance profile, box-ticking, clearance criteria, ability to perform sport-specific activity.
SP	‘Once they’re able to do these [sport-specific] activities, they need to do them in the environment they need to do them in, so to do them outside.’	Progression to outside, prioritise return to sport-specific and outdoor activities, relationship in sport-specific capabilities and environment, final stage to indoor activities.
SC1	‘But I think it needs to be quite a long process, you cannot go from inside to outside with there being much more injury risk.’	Return to outdoors, duration, longer phase transition, transition to outdoors, control over injury risk, reduce phase transition injury risk, managing phase transition, increment injury-risk exposure.
SC1	‘I think them being able to visualise something and react to it [in VR], being able to react to a stimulus, is good preparation for going then outside into a more chaotic football training session.’	VR visualisation tool, visualised stimuli reaction, scenarios, control visual scenarios, preparation for outdoors, VR preparation for chaotic training, VR incrementing chaos, VR transitioning outdoor participation, controlling for unpredictability.
SC2	‘So there is a short period just before they are going outside where they can start to do some of these tasks and skills, but not quite good enough to be outdoors. You’ve almost got them in a controlled environment. Getting them running and very easy change of direction work; to have them in VR might help them feel a little bit nicer to be outdoors.’	Last stage of indoor rehabilitation, prior to outdoor training, transitioning location, VR aid transition to outdoors, increments of progression, controlled environment indoors, VR running and change of direction, running, cutting, controlled transition of tasks, VR may aid ‘feeling a bit nicer to be outdoors’, psychological impact, mental confidence, VR for mental preparedness, VR for mental transition, functional tasks indoor and outdoor, simulating conditions, simulating environmental demand.
SC3	‘So really build it [return to cutting manoeuvres] up gradually in phases. So indoors it is a very predictable environment, you know that the surface is constant, you know you are not affected by wind, rain, wet, frost, ice, all that you can predict very strongly.’	Incremental phases to return to cutting, cutting, controlled progression, incremental return to sport, indoors is predictable, conditions during return to outdoors, control over weather indoors, control over environment during initial return to sport, safer return to outdoors, predictable factors inside, patients safe from outdoor elements, indoor versus outdoor surfaces.
SC3	‘[In VR] a predictable environment in that sense where everything is controlled, the player feels in charge of their environment. If it happened, let’s say in a contact injury, they might be scared of receiving the ball in that same position, or being taken out by another opponent, so maybe just keep it very predictable, and essentially in a zone that they can control.’	VR offers predictable environment, controlled environment, comfort, psychological benefit, participant control over environmental factors, fear of reinjury, fear of injury causing scenario, fear of return to play, safety, allows for confidence building, scenarios.

Codes were assigned deductively and systematically. Remaining data was coded inductively, open to different perspectives.

### 3.3.3 Example from secondary coder.

See Table 4 for an example of two (out of twelve) randomly selected data excerpts coded (blind) by the secondary coder with experience in qualitative research. These were discussed with the secondary coder alongside initial category ideas. To provide an indicator of inter-coder reliability, the Miles and Huberman (1994) [19] percentage agreement formula was applied: reliability = number of agreements/ (number of agreements + disagreements). Agreements and disagreements between coders were tallied for each extract. Codes that were similar in concept but phrased differently were considered an agreement between coders; additionally, multiple codes expressing the same concept were classed as one similarity [20]. Applying the formula gave the fractional percent of codes that agree, making it easy to compare to a desired agreement. For the coded extracts, the reliability percentage was 73%. Placing this into context, this is slightly less than the 80% minimum suggested by McAlister et al. [20].

**Table 4:** Coding example from secondary coder, SC = Strength and Conditioner.

#/12	Raw Data Extract	Corresponding Initial code
#5	‘a short period just before they are going outside where they can start to do some of these tasks and skills, but not quite good enough to be outdoors, you’ve almost got them in a controlled environment. Getting them running and very easy change of direction work; to have them in VR might help them feel a little bit nicer to be outdoors.’ SC2	Preparation, Final stages of rehabilitation, Positive impact of VR, Physio/Doctor perspective. ‘feel a little nicer’ – code on own as Psychological/Enjoyment/, Preparation for real-world.
#9	‘I think them being able to visualise something and react to it [in VR], being able to react to a stimulus, is good preparation for going then outside into a more chaotic football training session.’ SC1	Realistic, Scenario, Preparation for real-world, Player perspective.

### 3.3.4 Develop working analytical framework.

After all data was coded by line, the analytical framework was developed. All data that did not fit was coded as ‘other’. Table 5 presents part of the initial analytical framework: the interviewee is in the left-hand column, and the potential category is the heading ‘Return to outdoors’.

**Table 5:** Indexing: the collation and processing of codes into a developing thematic framework; SP = Sport Physiotherapist; SC = Strength and Conditioner.

Interviewee	Category: Return to outdoors
SP	Progression to outside, ankle is healthy, athlete can cope, functional state, clearance criteria, decisions, prioritise return to sport-specific and outdoor activities
SC1	Transition to outdoors, control/increment injury-risk exposure, VR visualisation tool, control visual scenarios, VR incrementing chaos, controlling unpredictability.
SC2	Prior to outdoor training, VR aid transition to outdoors, increments of progression, controlled environment indoors, VR for running and change of direction, controlled transition of tasks, VR for mental preparedness, functional tasks indoor and outdoor, simulating environmental demand.
SC3	Prior to outdoor activity, incremental, cutting, predictable factors inside, weather, surfaces, control over environment during initial return to sport, safer, VR predictable environment, controlled visual scenarios, comfort, psychological benefit, participant control, fear of injury-causing scenario, confidence-building.

This was eventually placed in the existing coding index or treated as emergent codes, expanding the coding index. As a result, the remaining analysis was more data-driven and inductive. For example, ‘When’/‘Timing of application’ was not on the list of interview topics asked of the rehabilitation specialists. However, grouped codes of similar and interrelated ideas were used as initial categories. In this instance, ‘Return to outdoors’ became an initial category. In this way, this potential category emerged from the interview responses.

Once all of the codes were collated into the thematic framework, there was a consistent revision of the original text, with the addition, adaptation, merging and deletion of codes.

In this example, there are four data sets, a small number. However, when there are more data sets, potentially hundreds, this process is essential to reduce information, aid clarity and allow for a coherent summary to be extracted.

### 3.3.5 Apply the analytical framework.

The codes were indexed by category, i.e., placed in the thematic framework (for demonstrative purposes; see Table 5 as an example that follows from the data set above).

After all codes were indexed, they were discussed and reviewed by two primary researchers until a coherent summary arose. See Table 6 for a summary of interviewees’ data for the category of ‘Time of VR application’. N.B. This was formed with reference to the full data set in an iterative process to ensure that accurate meaning was conveyed.

**Table 6:** Initial category of return to outdoors with refined codes.

Initial category	Codes
Return to outdoors	Joint is healthy/athlete can cope Prior to outdoor training Controlled transition of tasks

### 3.3.6 Chart data into framework matrix.

Data was summarised by category, first broadly (Table 7), and then in a more refined form with quotations (Table 8). These summaries were placed in a further spreadsheet matrix (Microsoft Excel).

**Table 7:** Summary of each interviewees’ data under specific category with overall summary; SP = Sport Physiotherapist; SC = Strength and Conditioner.

Interviewee	Time of VR application
SP	The initial goal is to return to a functional state. Prior, the body will not be able to cope with dynamic sporting demands. Once the joint is healthy and functionality is restored, sport-specific scenarios and a return to outdoor participation becomes the primary focus. Remaining or returning indoors would be regressive.
SC1	Phase transition from indoors to outdoors must be controlled, particularly increasing exposure to chaotic training. Incremented exposure to increasing demands could reduce re-injury risk. VR as a visualisation tool could allow for this level of preparation.
SC2	Just prior to outdoor training, athletes can perform functional running and simple cutting manoeuvres but have yet to transition to outdoor participation. This is a controlled environment. VR could aid the mental transition during this period by simulating environmental demand.
SC3	Incremental return to cutting is of focus and instigated indoors. Indoors is a predictable environment re: surface and weather. VR can offer a level of control so player feels in charge during injury-emulating scenarios. This may lead to fear reduction.
Summary	Dynamic activity should not be conducted until the body can cope (link to safety). Sport rehabilitation prioritises return to outdoor activity and sustained outdoor activity (link to monitoring functional capacity). Phase transition from indoor to outdoors is incremental (link to controlled progression). VR could provide control over scenarios and environment (link to safety and psychological benefits). VR prior to outdoor participation may involve running and incremented cutting manoeuvres (link to VR content). Conclusion: timing of VR use immediately prior to outdoor participation when functional and still indoors? VR scenarios to involve incremented running and cutting?

**Table 8:** Refined summary by subtheme with example quotations; SP = Sport Physiotherapist; SC = Strength and Conditioner.

Description of subtheme for category: ‘When: Time of VR Implementation’	Example quotations
The athlete should not use the VR application until their body can cope with its demands	‘During the early stages you just want it to be as easy as possible for them’ SC2 ‘You’re looking to get to a stage where you clear up all the basics, so you make sure you have what we term “the [joint] is healthy”: there is no swelling, there is no pain, there is full movement, there is isolated strength. And then once they’ve got that, and they are functionally day-to-day pain-free, we then move onto the sport-specific stuff’ SP
The VR application would only be used before the athlete transitions to outdoor participation	‘Once they’re able to do these [sport-specific] activities, they need to do them in the environment they need to do them in, so to do them outside’ SP
The VR application would be used to aid transition to outdoor participation	‘There is a short period just before they’re going outside when they can start to do some of these tasks or skills, but not quite good enough to be outdoors… Getting them running with very easy change of direction work, to have them in VR might help them’ SC2
The VR application would be used to provide a highly controlled environment	‘it needs to be quite a long process, you cannot go from inside to outside and there being a lot more injury risk.’ SC1 ‘you’ve got them in a controlled environment.’ SC2

### 3.3.7 Interpret the data.

**Fig 4:**
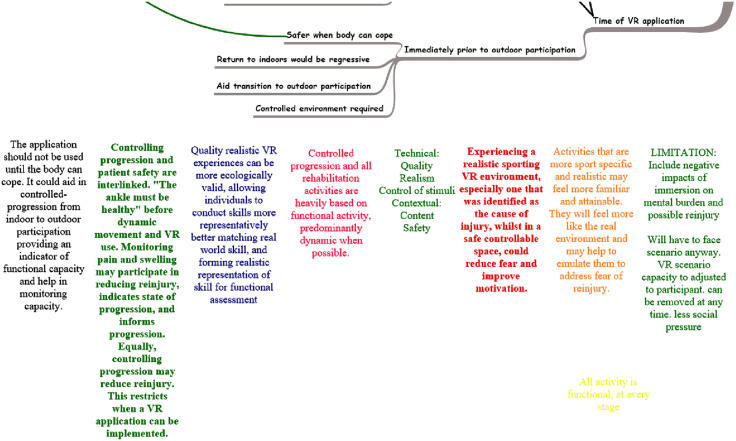
Developed categories mapped with linking and dividing concepts for discussion.

As characteristics and differences of data were identified to form themes, categories were developed and mapped on Inspiration 10, and a section of this map relating to the theme of ‘When’ can be seen in Fig 4. This process not only highlighted distinct categories, but also the links within and between these developed categories. The purpose of this diagram is to present the mind map in its working state. At this time of processing, the connections between themes and subthemes were still being developed.

### 3.3.8 Participant checking and auditing.

Dialogue was maintained with interviewees and representative quotations were approved. Ideas were discussed with an auditing biomechanist and an osteopath to review potential themes. This involved drawing on their existing knowledge regarding human movement and rehabilitation to remove experimenter bias and address topics in which the primary researcher would not otherwise have knowledge or experience.

### 3.3.9 Refine and process to convey results.

**Fig 5:**
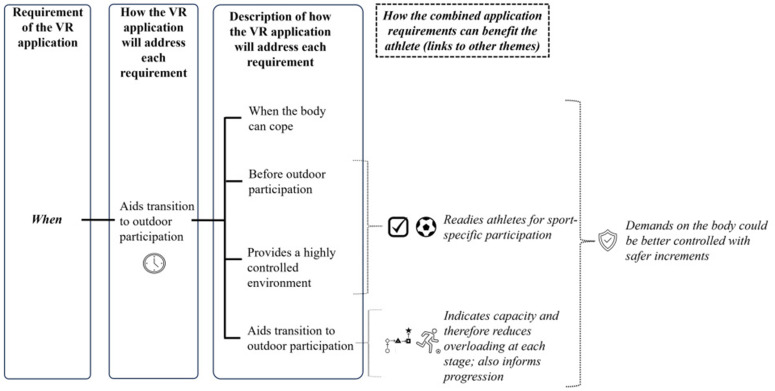
Theme and subthemes drawn in PowerPoint.

When a category was fully established, it was identified as a theme. The final theme of ‘When’ was identified and the following themes and subthemes were drawn on PowerPoint (Fig 5). The solidification of themes is best illustrated by the transition from the diagram in Fig 4, where categories were formed on Inspiration 10, to Fig 5, where the theme is shown in its final state drawn in PowerPoint.

Each diagram that presents a theme identifies a requirement of the VR application and how this will be specifically addressed to benefit the athlete. The sample data provided in this paper illustrates the evolution of the analytical process and demonstrates how the codes were refined into specific ways of meeting requirements. As a result, codes were categorised into themes that subsequently emerged as the overarching requirements.

The themes and subthemes are explained in full in a final dedicated publication. This worked example is a simplified version of one of the simpler themes. The theme of ‘When’ was less hierarchical in nature, with only one primary branch. For themes with more diverse subthemes, significant processing was required when developing the categories in order to better define the individual components.

## 4 Limitations, future work and conclusions

Gale et al. [10] identified the primary issues associated with the FA. For example, there are reflexivity, rigour and quality issues present in the use of the FA as with other qualitative methods. Additionally, although the tool itself is neither inductive nor deductive, the research may be impacted depending on how inductive or deductive the research question and researcher’s intentions are. The process is also time consuming. Additionally, the analysis should be overseen by an experienced researcher; however, Gale et al. [10] highlighted that this does not preclude those new to qualitative research from participating in the analytical process.

The study involved a limited number of participants, recruited through convenience sampling, resulting in a relatively niche cohort. Participants were selected based on their professional roles and place of employment, specifically as practising rehabilitation specialists working with athletes at a national level. Given their shared context, participants may have held similar perspectives, potentially limiting the diversity of viewpoints represented. This likely resulted in potential biases in practitioner perspectives, in particular, the focus on football in elite athletes, with experience in the treatment of injuries common to football players of this level being overrepresented in the data. Moreover, the advanced equipment available in national-level settings may not be accessible in community or local sporting environments, further constraining the transferability of findings. To enhance generalisability, future research could incorporate the perspectives of rehabilitation specialists operating in other contexts, as well as those of athletes themselves.

The reliability calculated as a percentage was slightly lower than that advised [20]. This may indicate that between the coders, the coding was not ideally consistent. Slight variations may have resulted from differences between coders as opposed to interviewees’ responses; however, the data may have been open to multiple interpretations. As the percentage was still relatively high at 73%, the threat to validity may not undermine the findings. However, the results should be read taking this into consideration.

The protocol could be validated with larger, more diverse samples, for example, those with contrasting perspectives from different practitioner roles, individuals that do not work for the same organisation, and athletes as patients and potential headset users. In combination, physiotherapists can contribute expert input during the design phase, with their interviews systematically transcribed and content-analysed to guide the development of clinically grounded VR systems. The dual practitioner-patient perspective could underscore the promise of VR for rehabilitation while also drawing attention to the methodological diversity and challenges that must be addressed for effective implementation in sport contexts.

Future work could integrate experiential insights from FA with established usability models and standards to enable a more comprehensive evaluation of VR applications. Once end users such as clinicians or athletes engage with the system, Nielsen’s heuristics [21] may be applied as a diagnostic tool to assess adherence to established design principles (e.g., visibility of system status, consistency, error prevention), or a modified ISO 9241−11 [22] could provide a complementary measurement model that defines usability in terms of effectiveness, efficiency, and satisfaction within a specified context of use. In a proof-of-concept study, post-task reflections could be collected using a ‘System Usability Scale’ [23] that adapts these heuristics for VR and captures dimensions such as psychological and emotional fidelity, spatial awareness, interaction quality, presence, and decision-making, such as the example seen in Table 9. Alongside these reflective measures, real-time user experiences could be captured through a ‘think-aloud’ protocol [24], where user-related events can be reported in real time, enabling participants to verbalise thoughts and difficulties during interaction, thereby yielding immediate insights into decision-making processes and emerging usability issues. Integrating quantitative usability metrics into the findings could offer a fuller perspective into application strengths, limitations and development.

**Table 9:** Potential System Usability Scale.

		Strongly disagree			Strongly agree	
		1	2	3	4	5
**#**	**Question**					
1.	I would like to use this VR application again.					
2.	The virtual room looked very different from the physical room.					
3.	The objects in the virtual room appeared to be the correct size.					
4.	The objects in the virtual room were positioned differently than in the physical room.					
5.	Moving in the virtual room did not make me feel sick or dizzy.					
6.	I felt less confident jogging or running in the virtual room than in the physical room.					
7.	I felt confident changing direction in the virtual room.					
8.	Changing direction using the arrow cues felt different compared to the physical world.					
9.	Moving around the avatar felt more like a real-life situation.					
10.	Changing direction around the arrows was more difficult than around the avatar.					

## Supporting information

S1 File Step-by-step protocol, also available on protocols.io. (PDF)
