# Supplementary material for: Lab protocol using framework analysis to capture practitioners’ perspectives on the usability of virtual reality for sport rehabilitation
Source: PLoS One. 2025 Dec 30;20(12):e0337814. doi: 10.1371/journal.pone.0337814 (PMC12752965; doi:10.1371/journal.pone.0337814)
Supplement: S1 File — (PDF) [file pone.0337814.s001.pdf]

Nov 04, 2025

Lab protocol using Framework Analysis to capture practitioners' perspectives on the usability of virtual reality for sport rehabilitation

In 1 collection

DOI
dx.doi.org/10.17504/protocols.io.e6nvw8r82vmk/v1

Hannah K.M. Tang[1], Mark J. Lake[1], Frederic A. Bezombes[1]
[1]Liverpool John Moores University

VR scenarios for assessi...

Hannah Tang
Liverpool John Moores University

OPEN ACCESS

DOI: **https://dx.doi.org/10.17504/protocols.io.e6nvw8r82vmk/v1**

Protocol Citation: Hannah K.M. Tang, Mark J. Lake, Frederic A. Bezombes 2025. Lab protocol using Framework Analysis to capture practitioners' perspectives on the usability of virtual reality for sport rehabilitation. **protocols.io** **https://dx.doi.org/10.17504/protocols.io.e6nvw8r82vmk/v1**

License: This is an open access protocol distributed under the terms of the **Creative Commons Attribution License**, which permits unrestricted use, distribution, and reproduction in any medium, provided the original author and source are credited

Protocol status: Working
We use this protocol and it's working

Created: April 21, 2025

Last Modified: November 04, 2025

Protocol Integer ID: 163862

Keywords: Framework analysis, Sport rehabilitation, Virtual reality , virtual reality for sport rehabilitation addressing issue, sport rehabilitation with technology, sport rehabilitation addressing issue, sport rehabilitation, vr application, vr feature, qualitative data analysis method, following vr application, aiding athlete rehabilitation, usability of virtual reality, athlete rehabilitation, better sport rehabilitation, rehabilitation process, perspectives on vr, existing rehabilitation practice, use of the framework analysis, vr, using framework analysis, virtual reality, qualitative enquiry, qualitative data processing, rehabilitation practice, present in all qualitative data processing, framework analysis, usability, technology feature, perspectives on the usability, based application, using technology, nursing research, sport, use of the current lab protocol, lab protocol, technology

Funders Acknowledgements:
Liverpool John Moores University PhD funding
Grant ID: Student ID 854667

Abstract

Addressing issues in sport rehabilitation with technology-based applications is becoming more common due to improved technological solutions. This drives the need for evidence-based rationales and decisions when it comes to technology features.
The current lab protocol paper proposes the use of the framework analysis (FA), a qualitative data analysis method, for identifying practitioners' perspectives on technology solutions aimed at aiding athlete rehabilitation. The FA allows data to be analysed in a structured and rigorous process, whilst also allowing for the flexibility associated with qualitative enquiry. Subsequently, this
method is being increasingly used in healthcare and nursing research and could be applied in the context of using technology to better sport rehabilitation. In the current paper, FA is applied in the context of determining the usability of virtual reality (VR) in sport rehabilitation and obtaining perspectives on VR features necessary for integration into existing rehabilitation practice. The paper includes a worked example, taking it from raw data to a working theme. The use of the current lab protocol led to the identification of interconnected key themes regarding the following VR application features: how the VR application would be delivered, what the VR application would involve, where the VR application would be used, and when the VR application would be used in the rehabilitation process. The lab protocol also allowed subthemes to be derived, indicating how these VR features would be met. The findings inform the ongoing development of a VR application designed to assess quick directional change in sports, potentially applied to the treatment of musculoskeletal injury in athletes. The use of the FA to derive this content is susceptible to limitations present in all qualitative data processing, such as reflexivity, the implications of levels of rigour, as well as being a time-consuming process.

Troubleshooting

Before start

Consolidated Criteria for Reporting Qualitative Research

This protocol is reported in accordance with the reporting guidelines of the ‘consolidated criteria for reporting qualitative research’ (COREQ) checklist https://academic.oup.com/intqhc/article/19/6/349/1791966 [1] (see Table 1). Each stage of the protocol relates to an item on the COREQ checklist.

Table 1 Consolidated criteria for reporting qualitative studies (COREQ): 32-item checklist

No	Item	Guide questions/description
**Domain 1: Research team and reflexivity**		
Personal Characteristics		
1.	Interviewer/facilitator	Which author/s conducted the interview or focus group?
2.	Credentials	What were the researcher's credentials? *E.g. PhD, MD*
3.	Occupation	What was their occupation at the time of the study?
4.	Gender	Was the researcher male or female?
5.	Experience and training	What experience or training did the researcher have?
Relationship with participants		
6.	Relationship established	Was a relationship established prior to study commencement?
7.	Participant knowledge of the interviewer	What did the participants know about the researcher? e.g. *personal goals, reasons for doing the research*
8.	Interviewer characteristics	What characteristics were reported about the interviewer/facilitator? e.g. *Bias, assumptions, reasons and interests in the research topic*
**Domain 2: study design**		
Theoretical framework		
9.	Methodological orientation and Theory	What methodological orientation was stated to underpin the study? *e.g. grounded theory, discourse analysis, ethnography, phenomenology, content analysis*
Participant selection		
10.	Sampling	How were participants selected? *e.g. purposive, convenience, consecutive, snowball*
11.	Method of approach	How were participants approached? e.g. *face-to-face, telephone, mail, email*
12.	Sample size	How many participants were in the study?
13.	Non-participation	How many people refused to participate or dropped out? Reasons?
Setting		
14.	Setting of data collection	Where was the data collected? e.g. *home, clinic, workplace*
15.	Presence of non-participants	Was anyone else present besides the participants and researchers?
16.	Description of sample	What are the important characteristics of the sample? *e.g. demographic data, date*
Data collection		
17.	Interview guide	Were questions, prompts, guides provided by the authors? Was it pilot tested?
18.	Repeat interviews	Were repeat interviews carried out? If yes, how many?
19.	Audio/visual recording	Did the research use audio or visual recording to collect the data?
20.	Field notes	Were field notes made during and/or after the interview or focus group?
21.	Duration	What was the duration of the interviews or focus group?
22.	Data saturation	Was data saturation discussed?
23.	Transcripts returned	Were transcripts returned to participants for comment and/or correction?
**Domain 3: analysis and findingsz**		
Data analysis		
24.	Number of data coders	How many data coders coded the data?
25.	Description of the coding tree	Did authors provide a description of the coding tree?
26.	Derivation of themes	Were themes identified in advance or derived from the data?
27.	Software	What software, if applicable, was used to manage the data?
28.	Participant checking	Did participants provide feedback on the findings?
Reporting		
29.	Quotations presented	Were participant quotations presented to illustrate the themes / findings? Was each quotation identified? e.g. *participant number*
30.	Data and findings consistent	Was there consistency between the data presented and the findings?
31.	Clarity of major themes	Were major themes clearly presented in the findings?
32.	Clarity of minor themes	Is there a description of diverse cases or discussion of minor themes?

The first two domains on the COREQ checklist provide study-specific details regarding the observations, interviews, and focus group. These are detailed in the documents below:

Domain 1: Research team and reflexivity (item number 1-8):

Research team and reflexivity.docx 23KB

Domain 2: Study design (item number 9-23):

Study design.docx

The following protocol contains a worked example using Framework Analysis to capture practitioners' perspectives on the usability of virtual reality for sport rehabilitation.

Domain 3: Analysis and Findings

1 **Data analysis**

24. Number of data coders: There was only one primary data coder who conducted all stages of data processing. However, one other individual with experience in qualitative research and data processing coded 12 randomly selected data excerpts (blind to the primary coding) for auditing processes. No major discrepancies were found and variations of codes were discussed for understanding and agreement.

2

Note

Definitions as applied in the context of this worked example [3]:

Analytical framework: A set of codes organised into categories used to organise the data, potentially arranged in a tree diagram structure. The framework creates a structure for the data that is helpful to summarise/reduce the data to answer the research question(s).
Categories: Codes are grouped into clusters around similar and interrelated ideas or concepts to help form categories, which are closely and explicitly linked to the raw data.
Code: A descriptive or conceptual label assigned to excerpts of raw data in a process called 'coding'.
Data: Texts, produced by transcribing interview data or creating 'field' notes while conducting participant-observation or observing objects or social situations.
Indexing: The systematic application of codes from the analytical framework to the whole dataset.
Matrix: A spreadsheet containing numerous cells into which summarised data are entered by codes (columns) and participants (rows).
NVivo: Qualitative data analysis software tool that allows, amongst other features, for data to be organised, coded, and categorised with ease.
Themes: Interpretive concepts or propositions that describe or explain aspects of the data, which are the final output of the analysis of the whole dataset. Themes are articulated and developed by interrogating data categories through comparison between and within participants. Usually, a number of categories would fall under each theme or sub-theme.
Thematic network: Mind-map-like illustrations that summarise the main themes.
Transcript: A written word-for-word account of an interview.

25. Description of the coding tree: This document describes the stages of a worked example, identified in Tables 5. The process is recursive and iterative and there is a lot of movement between stages. Activities listed in Table 5 have been added to the description in bullet point form for clarity.

Table 5. Stages to the data analysis process.

	A	B	C
	**Stage of data processing**	**Purpose of stage**	**Activity**
	A. Transcribe and familiarise	Record data in written form for initial processing and reviewing to increase familiarity.	Transcribe recording on Microsoft Word.
			Read transcripts and hand-written notes made during interviews. Check accuracy of transcripts.
			Write down ideas that are reflective and important on hand-written notes.
	B. Import to NVivo	Place data in analysis software tool for ease of processing.	Organise data by interviewee on NVivo.
	C. Assign initial codes	Break data down into smaller segments and assign labels (codes) for systematic comparison between datasets.	Identify initial expected codes, via deductive coding, using research question, interview topics and important ideas noted during and after interviews on NVivo.
			Assign deductive codes to all data systematically on Nvivo.
			Code data inductively on NVivo, open to as many different perspectives of end users as possible, e.g. athletes, exercise physiotherapists, strength and conditioners, etc.
			∗Second coder, with experience in qualitative research, blind codes 12 randomly selected data excerpts inductively on Microsoft Excel. Primary researcher and second coder discuss initial and potential codes with rationale and justifications.
	D. Develop working analytical framework	Identify similarities between codes and place into groups accordingly. This allows for further organisation of data into an initial analytical framework.	Form initial analytical framework by grouping codes into categories on NVivo. In this way, categories are defined.
			Label data that is not easy to categorise as 'other'.
	E. Apply the analytical framework	Place codes for all transcripts into the developing analytical framework (indexing) to solidify categories. Code any unlabelled	Apply the analytical framework by indexing transcripts, using existing categories and codes.
			Code all 'other' data, placing 'other' data into existing coding index and/or expanding

	A	B	C
		data to expand categories.	coding index with the emergent codes (Microsoft Excel).
	F. Chart data into the framework matrix	Summarise and tabulate data for later processing into themes. This facilitates the emergence of themes of more abstract concepts that represent recurring patterns or ideas across the coded data.	Summarise coded data by category with quotations, and place summaries in a further spreadsheet matrix (Microsoft Excel).
	G. Interpret the data	Develop and refine distinct themes, with visual mind-mapping representation.	Identify characteristics and differences of data to form themes.
			Refine themes to develop the thematic network map, using Inspiration 10 mind-mapping software.
	H. Participant checking and auditing	Ensure participant perspectives are accurately conveyed and underlying meanings are captured through suitable quotations and themes.	Maintain dialogue with interviewees and ensure representative quotations were approved by interviewees.
			∗Themes audited: discuss map and ideas with auditing biomechanist and osteopath to review themes.
	I. Refine and process to convey results	Finalise thematic network and separate to visually represent themes and their relationships, highlighting how they connect and interact with each other.	Separate thematic network by theme in order to transfer themes and subthemes from Inspiration 10 to PowerPoint and then to Microsoft Word. Attribute icons to represent links between themes. Tabulate information.
			∗Osteopath audited diagrams and table.
			Define and name themes. Researchers agree to finalise themes and subthemes.
			Hold a focus group to address diverse cases and subthemes in which the primary researcher would not otherwise have knowledge or experience.
			∗Check themes relate to coded extracts. Quotes audited against data set by language specialist.
			Finalised by research team. Paper produced.

∗Dependent on expertise available.

3 See protocol paper ‘Sample Data’ for example raw data and corresponding initial codes.

4 *Stage A) Transcribe and familiarise*:

- Transcribe recording on Microsoft Word.

The interviews were listened to and transcribed verbatim in Microsoft Word [3].

4.1
- Read transcripts and hand-written notes made during interviews. Check accuracy of transcripts.

The transcripts were then reviewed, being read alongside the contextual and reflective notes made during the interview [3] and checked for accuracy [2]. This increased familiarity with the content and provided an overview and insight into the breadth and depth of the results [3].

4.2
- Write down ideas that were reflective and important on hand-written notes.

Preliminary thoughts, especially regarding impressions, ideas, topics, and themes, were noted [5]. These initial analysing comments took into account prior knowledge of the literature and research field surrounding VR and rehabilitation [2]. This familiarisation provided the scope to contextualise the data and later aided analysis [3], particularly looking at any within- and between-participant differences. The initial ideas noted at this stage supported the development of the framework which was then constructed and described below [2].

5 *Stage B) Import to NVivo*:

- Organised data by interviewee on NVivo.

Transcript texts were exported and organised separately for each interviewee to the qualitative data analysis software tool NVivo Enterprise (QSR International, London, UK) [13].

6 *Stage C) Assign initial codes:*

- Identify initial expected codes, via deductive coding, using research question, interview topics, and important ideas noted during and after interviews on NVivo.

The use of the Framework Analysis was to address a strategic question in order to identify new plans and action [14]. Therefore, when reviewing the transcript data to generate the initial codes, this was primarily theory driven [2] and deductive [3], and the data was approached with the following three sources of information that were utilised to provide context and to provide parameters during initial analysis [14, 15]: 1) the research question, “What are the core features required for a VR application to enhance sport rehabilitation?”, 2) the list of interview topics based on themes that emerged from the literature review (see Table 6), and 3) the initially noted ideas that were made during the familiarisation stage.

Table 6. Combined topic list used during data analysis.

	A	B
	**No.**	**Interview topics**
	1	Injury background
	2	Treatment methods
	3	Return to sport
	4	Reinjury avoidance
	5	Sport-specific tasks
	6	Phases of rehabilitation and transitions between phases
	7	Return to outdoor activities
	**VR application**	
	10	VR features
	11	Feedback for the user from the VR application (both athlete and practitioners)
	12	Motivation within VR
	13	Specific examples of VR content suggested
	14	Overall thoughts on VR environment

6.1

- Assign deductive codes to all data systematically on NVivo.

Codes were allocated line by line and data was viewed as being split into smaller codable moments; however, a section may have been coded as one entire section, as well coded in smaller split-up data. The method referred to as “lumper” coding, in which several lines of data were coded as one, was conducted on an ad hoc basis at the discretion of the researcher [16], i.e. the entire transcripts were assessed by line (via NVivo) and generated systematically, with some of the surrounding data kept and reduced at a later point to retain context [2]. The construction of the initial codes was complex as the data set was rich.

Note

Codes were categorised, with each being assigned the same level of specificity and importance (a flat as opposed to hierarchal coding frame). There was no pre-established coding tree with existing branches to the initial categories.

6.2

- Code data inductively on NVivo, open to as many different perspectives of end users as possible, e.g. athletes, exercise physiotherapists, strength and conditioners, etc.

Note

This worked example uses a combined inductive-deductive approach. The coding is partly informed by the research question, interview questions (informed by literature review), and important noted ideas. However, the transcripts are also inductively coded, particularly to identify the categories of each subtheme/theme.

6.3

- Second coder, with experience in qualitative research, blind codes twelve randomly selected data excerpts inductively on Microsoft Excel. Primary researcher and second coder discussed initial and potential codes with rationale and justifications.

Twelve randomly selected data excerpts were coded (blind to the primary coding) by a second individual who has experience in qualitative research and data processing. Similarities and differences in coding were discussed, alongside implications for the codes used in the remainder of the data set.

7

D) Develop working analytical framework:

- Form initial analytical framework, by grouping codes into categories on NVivo. In this way, categories are defined.

The codes were then grouped into categories, which were then clearly defined by the process. This became the developing analytical framework. As a result of this, the codes were organised and data was managed with a new category-based structure [3].

7.1

- Label data that is not easy to categorise as ‘other’.

All data that was not easy to categorise was labelled as 'other' to avoid ignoring data that does not fit [3].

8 *E) Apply the analytical framework:*

- Apply the analytical framework by indexing transcripts on Microsoft Excel, using existing categories and codes.

The analytical framework acted as coding index, which was then used to label and organise all transcripts. The transcripts were indexed by line: selecting a section of data and assigning it an appropriate code within a category.

As coding progressed and similarities and differences of ideas emerged, categories developed [5]. Each emergent code was assessed as being a new potential category. Preliminary categories were grouped together into broader categories [5]. Subsequently, codes were collated across the entire data set [2]. This allowed data connections to be drawn between participants, as well as between answers to different questions in the same interview.

Note

This can be done by assigning numbers to codes and excerpts; however, in this worked example, these were actively cut and pasted into a table of categories. The data was exported from NVivo to the Excel spreadsheet and placed within the coding index.

Using Microsoft Excel provided a systematic layout and improved analysis as manipulating the position of codes relative to each other was simpler and connections and links could be easily identified.

8.1

- Code all 'other' data, placing 'other' data into existing coding index and/or expanding coding index with the emergent codes on Microsoft Excel.

As the interviews were semi-structured, additional topics emerged and were identified as further emergent codes. The remaining analysis was more data-driven [2] and inductive [3]. All other data was coded, placing 'other' data into existing coding index and/or expanding coding index with the emergent codes [3]. As a result, this extended the coding, and subsequently, there was a mix of expected codes (informed by the literature through interview topics) and emergent codes (informed solely by the data). No data was omitted from the coding process [3]. This framework was refined through an iterative process and until all transcripts coded and no further new codes emerged [3].

9 *F) Chart data into the framework matrix*

- Summarise coded data by category with quotations, and place summaries in a further spreadsheet matrix on Microsoft Excel.

The coded data was placed into a separate Excel spreadsheet matrix by category (columns) and participant (rows). Once data was tabulated by category, it was then

summarised with representative quotations [3]. This both reduced the data and allowed the data retain the original meaning of the interviewees' responses [3]. This table identified categories that developed into final themes for VR features.

10 **26. Derivation of the themes**:

Note

Summary of the development and application of the framework:
As described in point 25 (description of the coding tree), the use of the Framework Analysis was to address a strategic question in order to identify new plans and action [14]. Therefore, when reviewing the transcript data to generate the initial codes, this was primarily theory driven [2] and deductive. The interview topics (see Table 6) and initially noted ideas were used as expectant codes and were placed as listed headings in an Excel spreadsheet [5]. This became the working analytical framework, which was then used to label and organise all transcripts. However, as the interviews were semi-structured, additional topics emerged and were identified as further emergent codes. The remaining analysis was more data-driven [2] and inductive [3].

The themes were derived in the remaining stages of the data analysis process:

10.1 *G) Interpret the data*

- Identify characteristics and differences of data to form themes working between the original transcripts, notes, NVivo, and Microsoft Word and Excel.

As codes were collated into categories, recurrent ideas, patterns of meaning, and potential topics were extracted and organized under the overarching categories until the greater picture emerged [5]. This meant that characteristics and differences of data were identified, forming potential themes [3]. Data and codes were continually assessed in the context of the original transcripts [5]. Categories were then formed into themes of 'main VR application features' and eventually the sub-themes of 'the requirements needed to meet each feature'. Associations were made between themes and concepts, in a recursive process, until categories were refined, and themes, subthemes, and concepts were formed to a state suitable for mapping [2].

10.2

- Refine themes to develop the network map, using Inspiration 10 mind-mapping software.

The refinement of themes led to the development of a thematic network map [5]. The themes were mapped on a mind-mapping software, Inspiration 10. This allowed concepts to be connected, moved, grouped and divided due to visual linking and un-linking, which highlighted connections to explore relationships and causalities [3]. Mapping the data led to a greater solidification of main themes and sub-themes, whilst cross-referencing between the thematic framework and transcripts [2].

11 **27. Software**: Software was selected depending on what was fit for purpose: NVivo, Microsoft Word, Excel, PowerPoint, Inspiration 10.

12 **28. Participant checking**:

H) Participant checking and auditing

- Maintain dialogue with interviewees and ensure representative quotations are approved by interviewees.

The participants were emailed the quotations for approval and to add final thoughts. The extent of the feedback varied; however, the dialogue was concluded with the individuals stating they had no more comments to make. They were not provided with the findings after analysis. All further stages occurred without input from the interview participants.

Note

Final correspondence with the interview participants occurred on 25th Mar. 2021.

13 The following was conducted for auditing purposes:

- Audit themes: discuss map and ideas with auditing biomechanist and osteopath to review themes.

The thematic network map, with the themes (VR features) and subthemes (requirements to meet features), was then audited by a biomechanist specialising in sport and clinical biomechanics (PhD), as well as an osteopath (specialising in treating musculoskeletal injury). This involved discussing the entire thought process and rationale behind the analysis with the lead researcher to reduce knowledge-based limitations, which ensured pre-conceptions did not impact results.

All of the collated extracts were read for each theme to consider whether they appeared to form a coherent pattern. Those that did not fit were analysed to determine if this was due to the extract misplacement or poorly constructed themes [2]. All researchers agreed on finalised themes, subthemes and concepts.

Note

Auditing occurred at Liverpool John Moores University Byrom Campus on 26th Nov. 2021.

Reporting

14 **29. Quotations presented**: Participant quotations were presented to illustrate the themes and findings. Each quotation was identified with a participant number. The final data analysis stage allowed the results to be conveyed as clearly as possible:

15 **30/31/32. Data findings consistent/ Clarity of major and minor themes**:

Stage I) Refine and process to convey results.

Refining and processing the data to convey the results was performed in several stages:

- Separate thematic network by theme in order to transfer themes and subthemes from Inspiration 10 to PowerPoint and then to Microsoft Word. Attribute icons to represent links between themes. Tabulate information.

For reconstruction on PowerPoint, the thematic network map was separated by theme. This enabled a clear and manageable diagrammatic representation of themes and subthemes. This increased clarity; however, attention was needed to ensure that the thematic map was accurately transcribed in its entirety to the slides. This was achieved by representing themes with icons, which were placed where the link had existed, alongside an explanation on how the themes were linked and a description of the link.

VR features (themes) and requirements of each feature (subthemes) were placed in a global conceptual table and transferred to Microsoft Word. The advantages that stem from the links were also identified.

15.1

- Osteopath audited diagrams and table.

An osteopath familiar with treating musculoskeletal injuries audited the diagrams (both prior to and after the addition of icons), the description of connections within diagrams, and the tabulated information. This ensured that the presentation of the data was representative of the analysis process and outcomes.

Note

The above stage occurred on 22nd May 2022.

15.2

- Define and name themes. Researchers agree to finalise themes and subthemes.

The themes were named and defined, with the scope and content of each theme being distinct from one another [2]. The primary researcher, HT, finalised, defined and named topics for final review by the research team.

15.3

- Hold a focus group to address diverse cases and subthemes in which the primary researcher would not otherwise have knowledge or experience.

The focus group was held to discuss the findings. However, this was not part of the data analysis process. The focus group did not alter the results in any way and merely informed the discussion (see point 31 and 32 for rationale).

After the data analysis and the result were written, a focus group of eight participants was held at Liverpool John Moores University Byrom Campus to discuss the thematic network developed from the interview results. The rationale behind this was so that the discussion could address more diverse cases as well as subthemes in which the primary researcher would not otherwise have knowledge or experience.

Note

The focus group recruitment occurred on 26th May 2022. The focus group occurred on 31st May 2022.

Information sheets were provided and informed consent obtained. The focus group consisted of specialists with varied backgrounds and varying levels of VR experience, from novice to VR-rehabilitation researchers, as well as individuals with insight into the sport rehabilitation process, see Table 7.

Insights gained during the focus group further informed the discussion. The results section of the dedicated paper presents the findings of the interviews by theme; however, the discussion section ties both the interview results and the focus group discussion of the results together in a coherent narrative.

Table 7. Credentials of focus group participants.

	A	B
	**Participant**	**Relevant credentials**
	1	Professor of Clinical Biomechanics, research field: clinical gait analyst, expert in virtual rehabilitation
	2	Professor of Musculoskeletal Biomechanics, research field: mechanical properties of human muscles, tendons and joints in vivo
	3	Associate professor of Biomechanics, research field: musculoskeletal loading, injury and impairment in the lower limbs during sports activities
	4	Strength and Conditioning Coach
	5	Strength and Conditioning Coach, PhD candidate
	6	Biomechanist, PhD candidate
	7	Osteopath, biomechanist, PhD candidate
	8	Biomechanist (PhD), biomechanics technician

15.4 ▪ Check themes relate to coded extracts. Quotes audited against data set by language specialist.

Themes were assessed a final time, specifically to ensure they were applicable to both coded extracts and the full transcripts. The selection of quotations was audited by a specialist in English language to ensure that it was an accurate reflection of interviews, limiting selection and confirmation bias. The transcripts were read aloud by line and theme. Both the English language specialist and the primary researcher agreed on the meaning attributed to each concept through personal perspective (6th Jun. 2022). This was evaluated and consensus was established as to the meaning of the selected quotes, informed by context. Finally, interview data was listened to again, to ensure the most relevant quotations had been selected that best illustrated the themes.

15.5 ▪ Finalised by research team. Paper produced.

The sub-divided thematic network map and the corresponding table were finalised by the research team, and the overall concepts refined. The researchers agreed on the finalised themes, subthemes, and concepts.

A dedicated paper discusses the findings of the study.

Protocol references

References

1. Tong A, Sainsbury P, Craig J.
Consolidated criteria for reporting qualitative research (COREQ): a 32-item checklist for interviews and focus groups. International journal for quality in health care. 2007;19(6):349-57. doi: 10.1093/intqhc/mzm042.
2. Braun V, Clarke V. Using thematic analysis in psychology. Qualitative Research in Psychology. 2006;3(2):77-101. doi: 10.1191/1478088706qp063oa.
3. Gale NK, Heath G, Cameron E, Rashid S, Redwood S. Using the framework method for the analysis of qualitative data in multi-disciplinary health research. BMC Medical Research Methodology. 2013;13(1):1-8. doi: 10.1186/1471-2288-13-117.
4. DeJonckheere M, Vaughn LM. Semistructured interviewing in primary care research: a balance of relationship and rigour. Family Medicine and Community Health. 2019;7(2):e000057. doi: 10.1136/fmch-2018-000057.
5. Smith J, Firth J. Qualitative data analysis: the framework approach. Nurse Researcher. 2011;18(2):52-62. doi: 10.7748/nr2011.01.18.2.52.c8284.
6. Ritchie J, Lewis J. Qualitative research practice : a guide for social science students and researchers. London: SAGE; 2003.
7. Association TF. St George's Park 2025 [cited 2025 01/07]. Available from: https://www.englandfootball.com/england/St-Georges-Park.
8. Ardern CL, Glasgow P, Schneiders A, Witvrouw E, Clarsen B, Cools A, et al. 2016 Consensus statement on return to sport from the First World Congress in Sports Physical Therapy, Bern. British Journal of Sports Medicine. 2016;50(14):853-64. doi: 10.1136/bjsports-2016-096278.
9. Tassignon B, Verschueren J, Delahunt E, Smith M, Vicenzino B, Verhagen E, et al. Criteria-based return to sport decision-making following lateral ankle sprain injury: a systematic review and narrative synthesis. Sports Medicine. 2019;49(4):601-19. doi: 10.1007/s40279-019-01071-3.
10. Malanga GA, Yan N, Stark J. Mechanisms and efficacy of heat and cold therapies for musculoskeletal injury. Postgraduate medicine. 2015;127(1):57-65. doi: 10.1080/00325481.2015.992719.
11. Moc Králová D, Řezaninová J. The Role of Physiotherapists in Sport. 10th International Conference On Kinanthropology; September. Czech Republic2015.
12. Reiman MP, Lorenz DS. Integration of strength and conditioning principles into a rehabilitation program. International Journal of Sports Physical Therapy. 2011;6(3):241-53.
13. Alam MK. A systematic qualitative case study: questions, data collection, NVivo analysis and saturation. Qualitative Research in Organizations and Management: An International Journal. 2020;16(1):1-31. doi: 10.1108/QROM-09-2019-1825.
14. Parkinson S, Eatough V, Holmes J, Stapley E, Midgley N. Framework analysis: a worked example of a study exploring young people's experiences of depression. Qualitative Research in Psychology. 2016;13(2):109-29. doi: 10.1080/14780887.2015.1119228.
15. Hackett A, Strickland K. Using the framework approach to analyse qualitative data: a worked example. Nurse Researcher. 2018;26(2):8-13. doi: doi.org/10.7748/nr.2018.e1580.
16. Saldaña J. The coding manual for qualitative researchers. The coding manual for qualitative researchers. 2021:1-440.
